# From Virulence to Therapy: T6SS-Derived Antimicrobial Peptides A7 Combats APEC and MRSA Infections

**DOI:** 10.3390/ijms27073277

**Published:** 2026-04-04

**Authors:** Qin Lu, Zhaoran Zhang, Ziyi Zhang, Xiaodan Li, Chenchen Wang, Huanchun Chen, Qingping Luo, Chen Tan

**Affiliations:** 1 College of Veterinary Medicine, Huazhong Agricultural University, Wuhan 430070, China; luqin@webmail.hzau.edu.cn (Q.L.); zhaoran@mail.hzau.edu.cn (Z.Z.); zhangziyi@webmail.hzau.edu.cn (Z.Z.); 2018302110164@webmail.hzau.edu.cn (C.W.);; 2 Institute of Animal Husbandry and Veterinary, Hubei Academy of Agricultural Sciences, Wuhan 430064, China

**Keywords:** avian pathogenic *Escherichia coli* (APEC), methicillin-resistant *Staphylococcus aureus* (*MASA*), antimicrobial peptides (AMPs), type VI secretion system (T6SS), alternatives to antibiotics

## Abstract

The increasing prevalence of multidrug-resistant (MDR) pathogens, particularly avian pathogenic *Escherichia coli* (APEC) and methicillin-resistant *Staphylococcus aureus* (MRSA), poses a severe threat to the breeding industry and human health. To develop novel antibiotic alternatives, we adopted a “converting virulence into therapy” strategy by leveraging the type VI secretion system (T6SS) of the APEC strain ACN17-20. Guided by the structural analysis of T6SS Protein 00145, we rationally designed a series of amphipathic α-helical polypeptides. Among them, polypeptide A7 emerged as a lead candidate, exhibiting potent broad-spectrum antibacterial activity with negligible cytotoxicity against mammalian cells. Mechanistic studies revealed that A7 exerts a rapid bactericidal effect through a dual mode of action: physical disruption of bacterial membrane integrity leading to cytoplasmic leakage, and induction of lethal oxidative stress via reactive oxygen species (ROS) accumulation. Furthermore, A7 demonstrated excellent efficacy in eradicating pre-formed bacterial biofilms, addressing the challenge of persistent infections in breeding environments. In a mouse sepsis model induced by APEC and MRSA, A7 treatment significantly improved survival rates (60–80%), reduced bacterial loads in vital organs, and attenuated the systemic cytokine storm (*TNF-α* and *IL-1β*), thereby alleviating immune-mediated tissue damage. In conclusion, this study identifies polypeptide A7 as a safe therapeutic agent with a dual mechanism of action, providing a promising strategy to combat MDR infections and reduce antibiotic dependence.

## 1. Introduction

The rapid escalation of antimicrobial resistance (AMR) constitutes a critical threat to the sustainability of the global poultry industry and public health [1,2]. The emergence and dissemination of multidrug-resistant (MDR) pathogens, particularly avian pathogenic *Escherichia coli* (APEC), have severely compromised the efficacy of conventional antibiotics. APEC is the primary etiological agent of colibacillosis, a devastating disease causing air sacculitis, pericarditis, and septicemia in chickens, leading to significant economic losses due to mortality and carcass condemnation [3,4,5]. This crisis is exacerbated by the ability of *Escherichia coli* (*E. coli*) to form biofilms on farm equipment and water lines. Biofilms create physical barriers that render bacteria up to 1000-fold more resistant to antimicrobial agents and host immune defenses, resulting in persistent infections that are notoriously difficult to eradicate [6,7]. Consequently, there is an urgent imperative to develop novel, non-antibiotic antimicrobial agents with distinct mechanisms of action to ensure food safety and animal welfare [8].

Antimicrobial peptides (AMPs), essential components of the innate immune system, have garnered significant attention as promising alternatives to conventional antibiotics in poultry production [9]. Unlike traditional drugs that target specific metabolic enzymes, AMPs typically exert their bactericidal activity through rapid membrane disruption, a mechanism that imposes a high fitness cost for resistance development [10]. However, the agricultural application of native AMPs is often hindered by intrinsic limitations, including susceptibility to proteolytic degradation, potential toxicity, and reduced efficacy under physiological conditions [11,12]. To circumvent these obstacles, mining novel antimicrobial scaffolds from the bacterial competitive arsenal itself, coupled with structure-based rational design, has emerged as an innovative strategy [13].

The type VI secretion system (T6SS) is a contractile nanomachinery widely distributed in Gram-negative bacteria, including pathogenic *E. coli*. It functions as a “molecular crossbow” to inject toxic effector proteins into competitor cells, providing a survival advantage in polymicrobial environments such as the avian gut [14,15]. Having evolved to penetrate bacterial envelopes, T6SS effectors often possess intrinsic membrane-permeabilizing domains or cytotoxic motifs [16]. We hypothesized that these effectors harbor cryptic functional sequences that, through rational engineering, could be transformed into potent therapeutic peptides effective against poultry pathogens.

In this study, we mined the genome of ACN17-20, a clinically isolated APEC strain. We targeted its T6SS effector Protein 00145, guided by 3D structural analysis, identified a surface-exposed loop region as a progenitor template. Through rational modification—incorporating a hydrophobic core and optimizing charge distribution—we generated a series of engineered amphipathic α-helical peptides. Among them, peptide A7 demonstrated exceptional broad-spectrum potency against poultry-associated pathogens (including MDR *E. coli* and MRSA) and significant antibiofilm activity. Mechanistic investigations revealed that A7 acts via a dual mechanism involving membrane disruption and the induction of a lethal reactive oxygen species (ROS) burst. Furthermore, A7 exhibited high biosafety in mammalian cells and conferred significant protection in a murine sepsis model induced by the homologous APEC strain ACN17-17. This work presents a novel paradigm for discovering next-generation veterinary antimicrobials derived from the virulence factors of the pathogens themselves.

## 2. Results

### 2.1. Rational Design and Structural Characterization of Peptides Derived from Protein 00145

**Figure 1:**
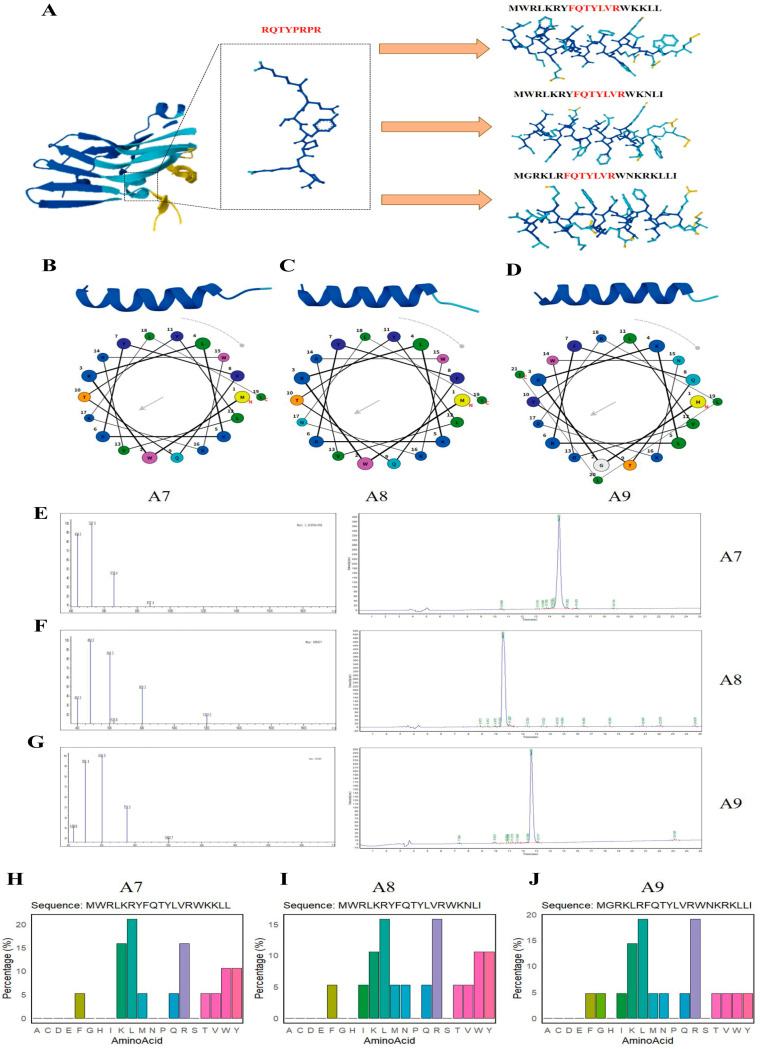
Rational design, structural characterization and validation of antimicrobial peptides based on the ACN17-20 T6SS Protein 00145. (**A**) Schematic diagram of the peptide engineering modification process. The loop region (RQTYPRPR) in the structure of Protein 00145 was optimized to generate amphipathic helical peptides. (**B**–**D**) Predicted three-dimensional models and helical wheel projections of peptides A7, A8 and A9, showing stable α-helical conformations with the characteristic segregation of hydrophobic and hydrophilic faces. (**E**–**G**) Chemical validation demonstrated that the mass spectra were consistent with the theoretical molecular weights, and the HPLC chromatograms (right) indicated a purity of >95%. (**H**–**J**) Amino acid composition distributions highlighted the enrichment of cationic (R, K) in diffrennt color and hydrophobic residues, confirming that the peptides possessed the physicochemical properties required for antimicrobial activity.

To develop novel antibacterial agents, a structure-based rational design strategy was applied to Protein 00145 of the type VI secretion system (T6SS) from *E.coli* ACN17-20. The solvent-accessible loop region in the native protein structure was selected as the initial template for peptide mining. The identified ancestral fragment (RQTYPRPR) was subsequently subjected to computer-aided optimization of physicochemical properties. The design process converted the disordered cyclic conformation into a stable amphipathic α-helix, a typical characteristic of potent antimicrobial peptides. This was achieved by replacing the native core sequence with a motif with enhanced hydrophobicity (FQTYLVR), and extending positively charged (e.g., arginine, lysine) and sterically hindered hydrophobic (e.g., tryptophan, phenylalanine) residues at the N- and C-termini (Figure 1A). Three resulting variants were designated as A7, A8 and A9, and their structural modeling revealed a distinct α-helical architecture, demonstrating that sequence modification successfully stabilized the secondary structure required for potential membrane permeabilization. To validate the effectiveness of the rational design strategy, we performed structural modeling and physicochemical property analysis on the three derived peptides (A7, A8 and A9). Secondary structure prediction indicated that all three variants adopted a well-defined α-helical architecture (upper panels), maintaining the structural stability of the template. Further analysis using helical wheel projections (lower panels) revealed that each peptide exhibited a prominent amphipathic topology. Hydrophobic residues (e.g., Leu, Phe, Trp) were highly concentrated on one side of the helix, forming a continuous hydrophobic face. In contrast, cationic and polar residues were segregated on the opposite side. This spatial arrangement generated a significant hydrophobic moment (indicated by the vector arrow), a key characteristic that facilitates the initial electrostatic attraction of peptides to anionic bacterial membranes and their subsequent insertion into the lipid bilayer (Figure 1B–D). Following rational design, the target peptides were chemically synthesized via solid-phase synthesis and subjected to rigorous purification. Electrospray ionization mass spectrometry (ESI-MS) and reversed-phase high-performance liquid chromatography (RP-HPLC) were employed to verify the identity and purity of the final products. The mass spectra confirmed that the observed molecular weights of A7, A8 and A9 were highly consistent with their respective theoretically calculated values. Furthermore, the HPLC chromatograms exhibited a single, sharp elution peak, indicating a high degree of homogeneity of the peptides with a purity exceeding 95%. These results ensured the structural integrity of the peptides used for subsequent biological assays (Figure 1E–G). To elucidate the physicochemical basis underlying the potential bioactivity of the peptides, we analyzed the amino acid composition of A7, A8 and A9. The composition profiles revealed a strategic balance between hydrophobicity and net positive charge. All three peptides were rich in cationic residues, predominantly arginine (R) and lysine (K), which accounted for a substantial proportion of the total sequences. This high density of cationic charge was designed to facilitate the initial electrostatic attraction between the peptides and the negatively charged bacterial cell envelope. Meanwhile, the incorporation of sterically hindered hydrophobic residues—such as leucine (L), phenylalanine (F) and tryptophan (W)—ensured sufficient hydrophobicity required for membrane insertion. The analysis confirmed the absence of acidic residues (aspartic acid or glutamic acid) in the sequences, thereby maximizing the net positive charge and minimizing electrostatic repulsion with the anionic bacterial membrane (Figure 1 H–J). The synthesized peptides demonstrated high aqueous solubility and were readily dissolved in PBS (PH = 7.4) without the need for special organic solvents.

### 2.2. Antimicrobial Activity Against Poultry-Associated Bacterial Models

**Figure 2:**
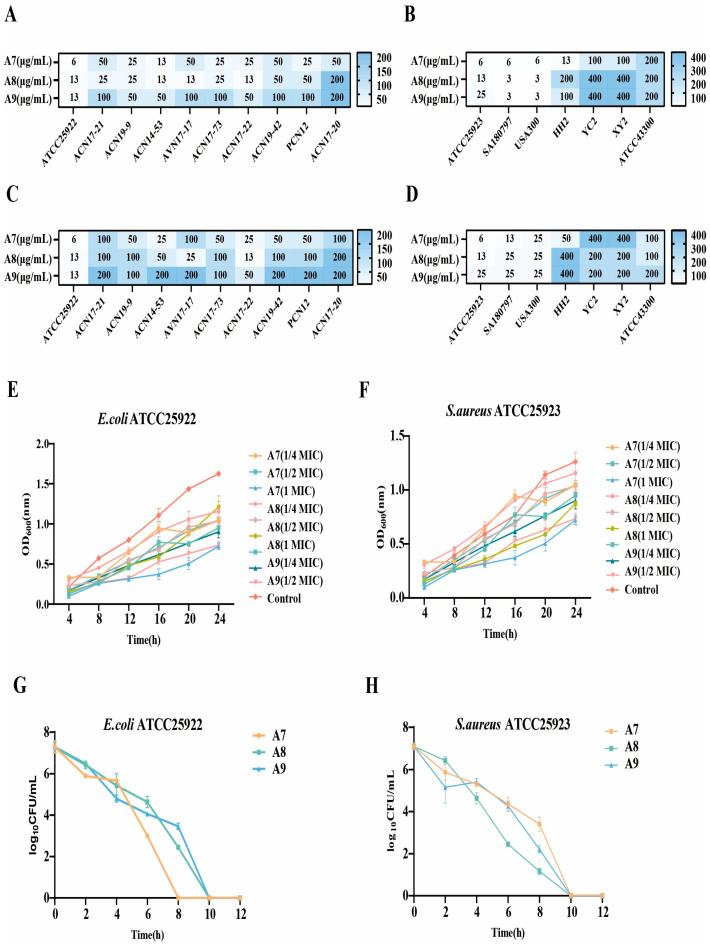
Quantitative evaluation of broad-spectrum antimicrobial potency and bactericidal kinetics. (**A**,**B**) Heatmap visualization of the antimicrobial susceptibility profiles. (**C**,**D**) The Minimum Inhibitory Concentrations and Minimum Bactericidal Concentrations were determined for peptides A7, A8, and A9 against a diverse library of pathogens. (**E**,**F**) Real-time growth inhibition kinetics monitored by optical density at 600 nm (OD_600_) over 24 h. *E. coli* ATCC25922 and *S. aureus* ATCC25923 were exposed to peptides at sub-inhibitory (1/4, ½ × MIC) and inhibitory (1 × MIC) concentrations. The curves demonstrate a dose-dependent suppression of bacterial proliferation compared to the untreated control. (**G**,**H**) Time-kill assays elucidating the rate of bactericidal action. The viability of *E. coli* and *S. aureus* (expressed as log10 CFU/mL) was quantified over a 10 h incubation period. A sharp, precipitous decline in CFU indicates rapid membrane permeabilization and cell death, with complete eradication achieved by 10 h for effective peptides.

To evaluate the potential of the designed peptides as novel antimicrobial candidates for the poultry industry, we determined the MIC and MBC values against a panel of representative pathogenic strains. The heatmap analysis reveals a distinct structure-activity relationship among the variants (Figure 2A–D). Peptide A7 exhibited superior potency against Gram-negative bacteria, particularly *E. coli* (Figure 2A), which is the primary etiological agent of avian colibacillosis. It displayed low MIC values (6–25 μg/mL) against the tested isolates. In contrast, peptide A8 demonstrated a marked preference for Gram-positive strains, showing exceptional efficacy against *S. aureus* (Figure 2B), a common cause of lameness and skeletal infections in broilers. The MICs for A8 ranged from 3 to 13 μg/mL against these strains. Critically, the MBC values closely mirrored the MICs (Figure 2C,D), indicating that these peptides exert a bactericidal rather than a bacteriostatic effect, a desirable trait for controlling bacterial load in poultry production environments. The dynamic impact of the peptides on the growth phases of avian pathogenic models was assessed via continuous OD_600_ monitoring. Peptide A7 induced a complete cessation of *E. coli* ATCC25922 growth at 8 μg/mL, maintaining the optical density at baseline levels throughout the 24 incubation (Figure 2E). Even at sub-inhibitory concentration (4 µg/mL), a significant extension of the lag phase was observed, suggesting a suppression of bacterial colonization potential. Similarly, for *S. aureus* ATCC25923 (Figure 2F), peptide A8 and A9 demonstrated the strongest inhibitory profile with clear dose-dependent stratification. This substantial suppression of turbidity at 13 µg/mL confirms the stability and sustained antimicrobial activity of the peptides, supporting their potential application in preventing bacterial proliferation in poultry. Rapid bacterial clearance is critical for minimizing disease transmission in flocks. To delineate the temporal efficacy of the peptides, time-kill assays were performed (Figure 2G,H). The results highlight a rapid mode of action characteristic of membrane-active peptides. Against *E. coli* (Figure 2G), all three peptides caused a swift reduction in viable cell counts, dropping from an initial load of 7.5 log10 CFU/mL to undetectable levels within 10 h. Notably, peptide A7 initiated a steep decline within the first 4 h, suggesting immediate interaction with the Gram-negative outer membrane. In the case of *S. aureus* (Figure 2H), peptide A8 and A9 exhibited the fastest killing rate, achieving a >3-log reduction (99.9% killing) within just 6 h. This distinct kinetic advantage of A8 and A9 validates its potential as a targeted agent for controlling Gram-positive infections in poultry.

### 2.3. Assessment of Hemolytic Activity, Cytotoxicity and Inhibition and Eradication of Bacterial Biofilms

**Figure 3:**
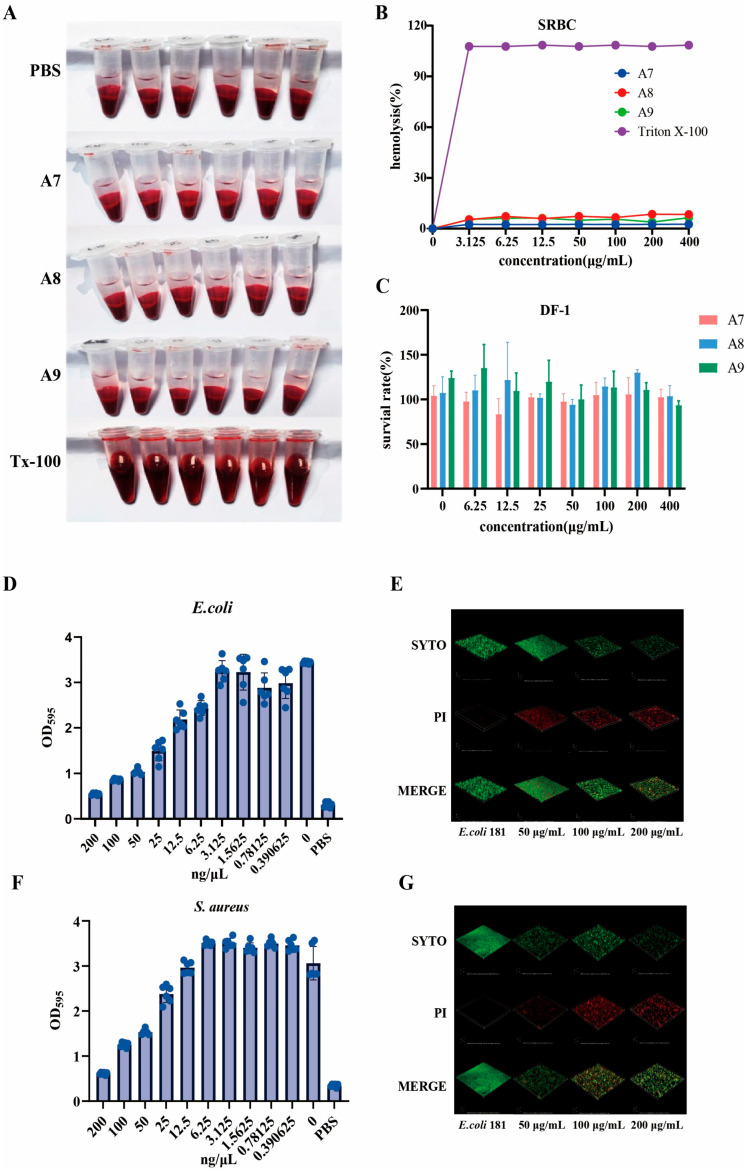
Evaluation of hemolytic activity and cytotoxicity of the engineered peptides. (**A**) Visual inspection of hemolysis in Sheep Red Blood Cells (SRBCs). Suspensions were incubated with peptides (A7, A8, A9) at varying concentrations. Phosphate-buffered saline (PBS) and 0.1% Triton X-100 served as negative and positive controls, respectively. (**B**) Quantitative analysis of hemolytic activity. The percentage of hemolysis was calculated relative to the Triton X-100 control (set as 100%). All three peptides exhibited negligible hemolytic effects (<5%) even at concentration (200 μg/mL). (**C**) Cytotoxicity assessment on DF-1 chicken embryo fibroblast cells. Cell viability was measured after treatment with peptides at concentrations (6.25 to 400 μg/mL). The results indicate high biocompatibility, with survival rates maintaining near 100% across the tested range. Data are presented as mean ± SD. (**D**) Quantification of *E. coli* biofilm biomass using the crystal violet staining assay. Established biofilms were treated with peptide A7 at indicated concentrations. The optical density at 595 nm (OD_595_) indicates the residual biofilm mass, demonstrating a dose-dependent eradication effect. (**E**) Visualization of *E. coli* biofilm architecture and bacterial viability using Confocal Laser Scanning Microscopy (CLSM). Biofilms were stained with SYTO9 and Propidium Iodide (PI) after treatment. The images reveal significant disruption of the biofilm matrix and extensive membrane damage at higher peptide concentrations (50–200 μg/mL). (**F**) Quantification of *S. aureus* USA300 biofilm biomass using the crystal violet staining assay. (**G**) Visualization of *S. aureus* USA300 biofilm architecture using CLSM after treatment, showing severe biofilm disruption and cell death at high concentrations.

To ascertain the potential of the engineered peptides for therapeutic application, their safety profiles were rigorously evaluated against mammalian and avian cells. First, the hemolytic activity was determined using Sheep Red Blood Cells (SRBCs). Incubation with peptides A7, A8, and A9 resulted in clear supernatants similar to the PBS negative control, indicating intact erythrocyte membranes (Figure 3A). In contrast, the positive control (Triton X-100) caused complete lysis, characterized by the release of hemoglobin. Quantitative analysis (Figure 3B) confirmed that all three peptides exhibited extremely low hemolytic activity. Even at a high concentration (200 μg/mL), the hemolysis rates remained below 10%, highlighting a significant selectivity for bacterial membranes over eukaryotic cell membranes. Furthermore, cytotoxicity was assessed using the DF-1 cell line (Figure 3C). The cell survival rates remained unaffected by peptide treatment, with viabilities exceeding 90% even at concentration (400 μg/mL) (significantly higher than the effective MIC values). These results demonstrate that the peptides possess excellent biocompatibility and a high therapeutic index, suggesting minimal risk of host tissue toxicity. Bacterial biofilms represent a significant challenge in clinical treatment due to their resistance to conventional antibiotics. Therefore, we evaluated the ability of peptide A7 to disrupt established biofilms of *E. coli* 181 and *S. aureus* USA300. Quantitative analysis using the crystal violet assay demonstrated that A7 significantly reduced biofilm biomass in a concentration-dependent manner (Figure 3D,F). As the peptide concentrations increased (0.39–200 μg/mL), a precipitous drop in OD_595_ was observed. Notably, at a concentration (200 μg/mL), the residual biofilm mass was comparable to the PBS control, suggesting near-complete eradication of the sessile communities for both Gram-negative and Gram-positive strains. To further investigate the microscopic mechanism, Confocal Laser Scanning Microscopy (CLSM) was employed with live/dead staining (Figure 3E,G). The untreated control groups exhibited dense, multi-layered architectures composed primarily of viable bacteria (intense green fluorescence). In contrast, treatment with A7 resulted in a progressive collapse of the 3D biofilm structure. At concentrations (100 μg/mL and 200 μg/mL), a dramatic shift from green to red fluorescence was observed, indicating that A7 successfully penetrated the biofilm matrix and compromised the membrane integrity of the embedded bacteria. These results confirm that A7 possesses potent antibiofilm properties, capable of both dispersing the biofilm matrix and killing the distinct bacteria.

### 2.4. Structure-Based Engineering of α-Helical Peptides from T6SS Protein 00145: Dual Mechanism of Membrane Disruption and ROS Induction Against Multidrug-Resistant Pathogens

**Figure 4:**
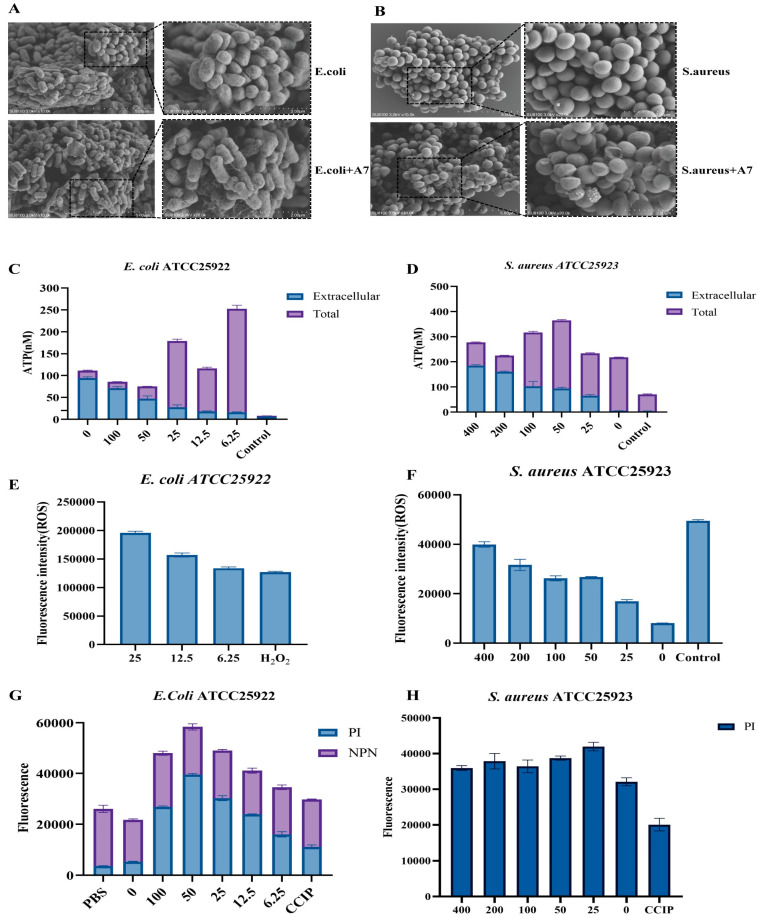
Elucidation of the bactericidal mechanism of peptide A7. (**A**,**B**) Scanning Electron Microscopy (SEM) images visualizing morphological alterations. *E. coli* ATCC25922 and *S. aureus* ATCC25923 were treated with peptide A7 (bottom panels) or PBS control (top panels). Treated cells exhibit severe membrane corrugation, pore formation, and leakage of cytoplasmic contents compared to the smooth, intact control cells. (**C**,**D**) Assessment of membrane permeability via ATP efflux. Extracellular and total ATP levels in *S. aureus* ATCC25923 and *E. coli* ATCC25922 were quantified after treatment, indicating concentration-dependent leakage of intracellular ATP. (**E**,**F**) Investigation of inner and outer membrane integrity. Uptake of Propidium Iodide (PI) in *S. aureus* ATCC25923 indicates compromised membrane integrity. Dual-dye assay using N-phenyl-1-naphthylamine (NPN) and PI in *E. coli* ATCC25922 confirms disruption of both the outer membrane (NPN uptake) and inner membrane (PI uptake). (**G**,**H**) Measurement of intracellular reactive oxygen species (ROS) generation. Fluorescence intensity reflects ROS accumulation in *S. aureus* ATCC25923 and *E. coli* ATCC25922. Peptide A7 treatment induces significant oxidative stress comparable to the H_2_O_2_ positive control. Data are presented as mean ± SD.

Visualization of Membrane Damage via SEM: To directly visualize the impact of peptide A7 on bacterial cell morphology, Scanning Electron Microscopy (SEM) was performed. As shown in Figure 4A,B, the untreated control cells displayed a smooth, bright, and plump surface architecture, indicating intact cell envelopes. In sharp contrast, cells exposed to A7 exhibited severe morphological aberrations. For *E. coli*, the cell surface appeared corrugated and collapsed, with visible deep crater-like disruptions (Figure 4A). Similarly, treated *S. aureus* cells showed distorted shapes and surface roughness, suggesting membrane lysis and the leakage of cytoplasmic contents (Figure 4B). These results provide direct evidence that A7 targets and physically disrupts the bacterial cell membrane. Compromise of Membrane Integrity and ATP Leakage: To corroborate the physical damage observed by SEM, we assessed the biochemical integrity of the membrane. Since ATP is crucial for cell viability, its leakage serves as a sensitive indicator of membrane perforation. Treatment with A7 led to a dose-dependent increase in extracellular ATP levels in both strains (Figure 4C,D), confirming that the peptide induces transmembrane pore formation. Furthermore, membrane permeability was probed using hydrophobic dyes (Figure 4E,F). In *E. coli*, A7 treatment triggered a significant uptake of NPN (an outer membrane probe) and PI (a DNA-binding dye that penetrates damaged inner membranes), demonstrating that the peptide effectively permeabilizes both the outer and inner membranes of Gram-negative bacteria (Figure 4F). Similarly, enhanced PI fluorescence was observed in *S. aureus*, indicating substantial membrane compromise (Figure 4E). Accumulation of Intracellular Reactive Oxygen Species (ROS): Beyond membrane disruption, we investigated whether A7 induces oxidative stress as a downstream bactericidal mechanism. Using the DCFH-DA fluorescent probe, we monitored intracellular ROS levels. As illustrated in Figure 4G,H, A7 treatment elicited a rapid and robust surge in ROS production in both *E. coli* and *S. aureus*. The fluorescence intensity at bactericidal concentrations was comparable to that of the hydrogen peroxide H_2_O_2_ positive control. This suggests that A7 not only physically damages the membrane but also disrupts cellular respiration or metabolism, leading to lethal oxidative stress.

### 2.5. Therapeutic Efficacy in a Mouse Model of ACN17-17 Systemic Infection

**Figure 5:**
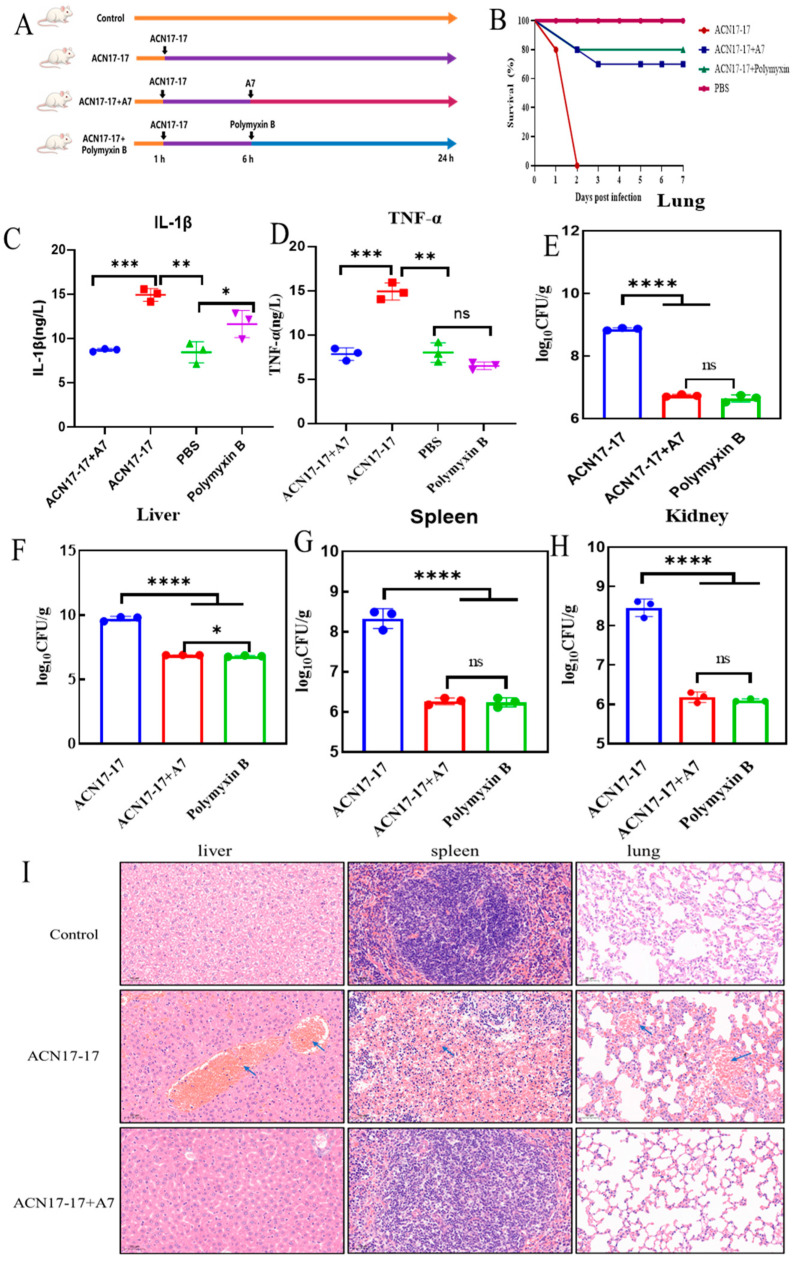
In vivo therapeutic efficacy of peptide A7 in a mouse model of ACN17-17 induced peritonitis. (**A**) Schematic representation of the experimental design. Mice were intraperitoneally challenged with lethal doses of ACN17-17. Treatment with peptide A7 or Polymyxin B (positive control) was administered 6 h post-infection. (**B**) Kaplan-Meier survival curves monitored over 7 days. Treatment with A7 significantly improved survival rates compared to the untreated infection group (ACN17-17), which showed 100% mortality within 2 days. (**C**,**D**) Analysis of pro-inflammatory cytokines in serum. The levels of IL-1β and TNF-α were quantified by ELISA. A7 treatment significantly attenuated the cytokine storm induced by bacterial infection. (**E**–**H**) Assessment of bacterial burden in major organs. Bacterial counts log10 CFU/g) in the Lung, Spleen, Kidney, and Liver were determined. Peptide A7 significantly reduced bacterial colonization in all tested tissues, comparable to the antibiotic control. (**I**) Histopathological evaluation of organ tissues (Liver, Spleen, Lung) via H&E staining. The untreated group (ACN17-17) displayed severe tissue necrosis, inflammatory cell infiltration, and structural disarray, while the A7-treated group exhibited marked alleviation of pathological damage, resembling the healthy control. Data are presented as mean ± SD (ns, not significant, * *p* < 0.05, ** *p* < 0.01, *** *p* < 0.001, **** *p* < 0.0001).

To evaluate the clinical potential of peptide A7, we established a mouse model of acute systemic infection induced by the ACN17-17 strain (Figure 5A). As shown in the survival analysis (Figure 5B), the untreated group succumbed to the infection rapidly, with a 100% mortality rate observed within 48 h. In stark contrast, a single dose of peptide A7 administered 6 h post-infection significantly rescued the mice, resulting in a survival rate comparable to that of the potent antibiotic Polymyxin B. Sepsis-related mortality is often driven by an excessive immune response, known as a cytokine storm. We therefore quantified serum inflammatory markers. Infection with ACN17-17 triggered a dramatic upregulation of IL-1β and TNF-α (Figure 5C,D). However, treatment with A7 effectively suppressed these pro-inflammatory cytokines, preventing the onset of hyper-inflammation. Furthermore, we assessed the bacterial burden in vital organs to evaluate the peptide’s ability to control systemic dissemination. As depicted in Figure 5E–H, high bacterial loads were recovered from the lung, spleen, kidney, and liver of untreated mice. Treatment with A7 resulted in a substantial reduction in bacterial counts (by several orders of magnitude) across all organs (*p* < 0.0001), confirming its potent bactericidal activity in vivo. Histological examination via H&E staining provided further evidence of organ protection (Figure 5I). Tissues from the infection group displayed classical signs of severe pathology, including massive inflammatory cell infiltration, alveolar thickening in the lungs, and necrotic lesions in the liver and spleen. Notably, mice treated with A7 exhibited preserved tissue architecture with minimal inflammation, demonstrating that the peptide effectively protects host tissues from infection-induced damage.

### 2.6. Evaluation of Anti-MRSA Efficacy In Vivo

**Figure 6:**
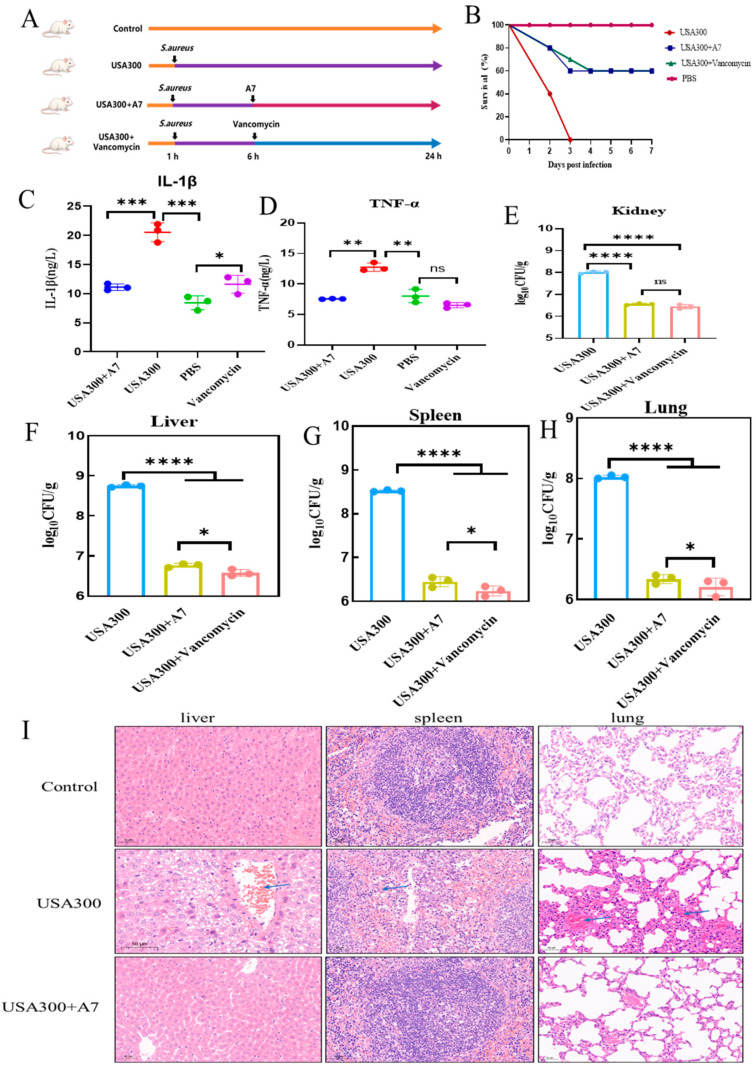
In vivo therapeutic efficacy of peptide A7 in a lethal murine model of MRSA (USA300) sepsis. (**A**) Schematic of the experimental protocol. Mice were intraperitoneally infected with *S. aureus* USA300. Peptide A7 or Vancomycin (positive control) was administered 6 h post-infection. (**B**) Kaplan–Meier survival analysis (*n* = 10). Treatment with A7 significantly rescued mice from lethal challenge, achieving a 60% survival rate compared to 0% in the untreated group. (**C**,**D**) Quantification of serum pro-inflammatory cytokines *TNF-α* and *IL-1β* by ELISA. A7 treatment effectively mitigated the systemic inflammatory response induced by MRSA infection. (**E**–**H**) Determination of bacterial burden in the Kidney, Liver, Spleen, and Lung. Data are expressed as log10 CFU/g. A7 significantly reduced bacterial colonization in all vital organs (*p* < 0.0001), comparable to the Vancomycin control. (**I**) Histopathological analysis via H&E staining. Representative images of the liver, spleen, and lung show severe inflammatory infiltration and tissue necrosis in the USA300 group, whereas A7 treatment preserved tissue architecture and alleviated pathological damage. Data are expressed as mean ± standard deviation (SD). Statistical significance is indicated as * *p* < 0.05, ** *p* < 0.01, *** *p* < 0.001, and **** *p* < 0.0001, ns, not significant.

Given the broad-spectrum activity of peptide A7, we further investigated its therapeutic efficacy in a lethal sepsis model induced by the multidrug-resistant *S. aureus* strain USA300 (Figure 6A). Survival monitoring revealed that the infection was highly virulent, causing 100% mortality in the untreated vehicle group within 3 days. However, a single therapeutic dose of A7 administered 6 h post-infection conferred a significant survival advantage, rescuing 60% of the mice (Figure 6B). This protective effect was comparable to that of vancomycin, the clinical drug of last resort for MRSA infections. To elucidate the physiological basis of protection, we analyzed the systemic immune response. USA300 infection provoked a robust cytokine storm, evidenced by elevated serum levels of TNF-α and IL-1β. Notably, treatment with A7 significantly downregulated these pro-inflammatory mediators to levels similar to the uninfected control (Figure 6C,D), thereby preventing lethal septic shock. Concurrently, we assessed the bacterial load in major organs to verify pathogen clearance. As shown in Figure 6E–H, untreated mice exhibited heavy bacterial colonization in the kidney, liver, spleen, and lung. Treatment with A7 resulted in a substantial reduction of bacterial burden by approximately 1.5 to 2 log units across all tested tissues, demonstrating the peptide’s capability to inhibit systemic dissemination of MRSA. Histological Protection of Vital Organs: The macroscopic protection was corroborated by histopathological evaluation (Figure 6I). In the untreated group, the lungs displayed alveolar thickening and congestion, while the liver and spleen showed extensive necrotic lesions and inflammatory cell infiltration. In contrast, tissues from A7-treated mice exhibited markedly alleviated pathological changes with preserved structural integrity, confirming that the peptide effectively mitigates infection-mediated organ damage.

## 3. Discussion

The growing antibiotic resistance (AMR) crisis in poultry farming urgently requires the discovery of novel therapeutic frameworks that go beyond traditional host defense peptides, which are often limited by low stability and high production costs [17]. In this study, we demonstrate a paradigm shift of “fighting fire with fire”—namely, mining antibacterial candidates from the type VI secretion system (T6SS) of *E. coli*. Recent advances in genome mining and artificial intelligence have demonstrated great potential of converting bacterial virulence factors into therapeutic agents [12,18]. Unlike traditional screening methods, our rational design strategy focuses on T6SS effector proteins, a molecular machinery evolutionarily optimized for interbacterial competition [16]. We engineered polypeptide A7 by identifying its cryptic pore-forming domain and enhancing its amphipathicity and cationic charge. This approach not only preserved the intrinsic membrane-penetrating ability of the T6SS effector protein but also significantly improved its solubility and specificity against avian pathogens, which is consistent with recent findings that modifying secretion system effector proteins can generate potent antimicrobial peptides [19]. By rationally enforcing the cryptic loop region to form an amphipathic α-helical structure, we endowed polypeptide A7 with enhanced structural stability and cationic density. This “from virulence to therapy” conversion provides a robust paradigm for mining next-generation antimicrobials from the vast treasure trove of bacterial secretion systems [12].

A key advantage of polypeptide A7 over conventional antibiotics lies in its “double hit” mechanism, which imposes an extremely high fitness cost on the development of drug resistance. Our results demonstrated that A7 not only physically disrupts bacterial cell membranes but also triggers an intracellular burst of lethal reactive oxygen species (ROS) [20]. This is consistent with recent studies showing that ROS accumulation impairs bacterial metabolic homeostasis and DNA integrity, acting as the second major bactericidal driver following membrane permeabilization [21]. Biofilm formation on poultry farming equipment is a major cause of recurrent infections. A7 exhibits outstanding efficacy in eradicating pre-formed biofilms, a property that can be attributed to its ability to penetrate the extracellular polymeric substance (EPS) matrix and kill metabolically dormant cells. This antibiofilm activity outperforms that of many conventional antibiotics which cannot cross the EPS barrier, highlighting A7’s potential to address persistent infections in the poultry farming environment [7]. The bactericidal activity of polypeptide A7 is fundamentally governed by its physicochemical interactions with the bacterial envelope. Our mechanistic data (SEM, NPN/PI uptake, ATP leakage) strongly suggest that the mechanism of action of A7 conforms to the “carpet model” or the “toroidal pore model” [22]. The positively charged arginine/lysine residues of A7 facilitate its rapid accumulation on the negatively charged bacterial surface via electrostatic attraction, displacing divalent cations and disrupting the stability of the lipopolysaccharide (LPS) layer. Subsequently, the hydrophobic face of the amphipathic helix inserts into the lipid bilayer, inducing severe membrane curvature changes and the formation of transient pores [23]. This disruption causes the collapse of the transmembrane electrochemical potential (ΔΨ) [24] and triggers the irreversible efflux of critical cytoplasmic components (ATP) [25], ultimately leading to cell death. In contrast to receptor-mediated antibiotics, this non-enzymatic, physical mode of action that targets the fundamental lipid structures of bacteria renders the development of bacterial resistance functionally difficult and evolutionarily costly [19,26].

The clinical translation of antimicrobial peptides is often hindered by systemic toxicity and serum inactivation [11]. In our lethal sepsis model, polypeptide A7 exhibited therapeutic efficacy comparable to that of colistin B (against APECAPEC) and vancomycin (against MRSA)—the clinical “last-resort” antibiotics—achieving a significant survival rate (60–80%) and bacterial clearance. However, a cross-comparison revealed the unique advantages of A7. While colistin B is clinically limited by dose-dependent nephrotoxicity and neurotoxicity [27], A7 demonstrated high biosafety with no hemolytic or cytotoxic effects on mammalian cells, indicating a more favorable therapeutic index. Furthermore, unlike conventional antibiotics that, despite their potent bactericidal activity, may exacerbate septic shock due to massive endotoxin release [28], A7 exerted robust immunomodulatory properties. It significantly attenuated the systemic “cytokine storm” (TNF-α, IL-1β), a property that may be attributed to its ability to bind and neutralize LPS or inhibit pro-inflammatory signaling pathways [29,30]. This dual capacity—combining bactericidal clearance with immunopathological mitigation—renders A7 a more comprehensive candidate for the treatment of severe Gram-negative bacterial sepsis in poultry, while addressing both pathogen load and dysregulated host responses [31].

Despite the promising therapeutic efficacy demonstrated by peptide A7, this study has certain limitations that warrant further investigation. Our in vivo efficacy was primarily evaluated using a single homologous strain (ACN17-17) and a single MRSA strain (USA300). As indicated by our in vitro MIC/MBC assays, the susceptibility to A7 varies among different clinical isolates. Such strain-specific effects of antimicrobial peptides are well-documented in the literature, highlighting that subtle differences in bacterial membrane composition or metabolic state can significantly influence peptide efficacy [32,33,34]. Future studies should expand the in vivo evaluations to include a broader panel of clinical isolates to comprehensively assess the translational potential of A7 across diverse strain backgrounds.

## 4. Materials and Methods

### 4.1. Strains and Main Reagents

*E.coli* ATCC25922 and *Staphylococcus aureus* ATCC25923 were purchased from the China Institute of Veterinary Drug Control and preserved in our laboratory. Strains ACN17-20, ACN17-17 and USA300 were isolated and preserved in our laboratory.

Colistin was obtained from Solarbio Science & Technology Co., Ltd. (Beijing, China). SYTO 9 was purchased from Fosun Biotech Co., Ltd. (Shanghai, China). Propidium Iodide (PI) and 1-N-phenylnaphthylamine (NPN) were supplied by Biosharp Life Sciences(Beijing, China). ATP Assay Kit and reactive oxygen species (ROS) Assay Kit were purchased from Beyotime Institute of Biotechnology (Shanghai, China). Strains ACN17-20, ACN17-17 and USA300 were isolated and preserved in our laboratory. Bacteria were routinely cultured in Luria–Bertani (LB) at 37 °C with shaking at 220 rpm. For long-term storage, all strains were maintained as glycerol stocks (50% *v*/*v*) at −80 °C.

### 4.2. Composition and Schematic Mapping of T6SS Gene Cluster

The sequences of 13 conserved T6SS genes in *E. coli* were retrieved from the SecReT6 database to construct the gene schematic map. Phylogenetic analysis was performed based on the vgrG gene sequence and T6SS1, T6SS2, T6SS3 sequences from NCBI to identify the T6SS subtype of the strains. The phylogenetic tree of the vgrG gene from strain ACN17-20 was constructed by the neighbor-joining method using MEGA 6 software.

### 4.3. Structural Analysis and Prediction of Antimicrobial Peptides Encoded by Genes of Unknown Function

Homology modeling of the three-dimensional structure was conducted via SWISS-MODEL and Phyre2 servers. Secondary structural elements (e.g., α-helix, β-sheet) of the modified peptides were predicted usingthe online websites of JPre4 (http://www.compbio.dundee.ac.uk/jpred4, accessed on 1 April 2026) and PSIPRED (http://bioinf.cs.ucl.ac.uk/psipred/, accessed on 1 April 2026) tools. Key parameters including net charge, hydrophobicity, amphipathic moment and Boman index were calculated by the analytical tools of APD3 database for the analysis of antimicrobial peptide derivative sequences, and the optimal amino acid sequence was screened out. The target sequence was synthesized by GL Biochem (Shanghai, China) Co., Ltd.

### 4.4. Determination of Minimum Inhibitory Concentration (MIC) and Minimum Bactericidal Concentration (MBC)

MIC values of all compounds were determined by the standard broth microdilution method according to the Clinical and Laboratory Standards Institute (CLSI) guidelines. Briefly, the test antimicrobial peptides were dissolved in PBS to prepare a stock solution concentration (1000 μg/mL), which was then diluted with Mueller–Hinton Broth (MHB) to obtain the working solution. The working solution was two-fold serially diluted with MHB in a 96-well plate (100 μL final volume per well). Bacterial cells were washed three times with PBS and adjusted to a concentration of 1 × 10^5^ CFU/mL. A total of 100 μL of the bacterial suspension was added to each well and mixed with the peptide solution, followed by incubation at 37 °C for 24 h. The absorbance at 600 nm was measured using a microplate reader, with pure MHB as the negative control and untreated bacterial suspension as the positive control. Each compound was tested in three replicate wells, and the experiment was repeated at least three times. A total of 50 μL of the standard bacterial suspension was added to wells 1 to 11 and incubated at 37 °C for 16–20 h; MIC value was determined by observing bacterial growth. The bacterial suspension from each well was spread on MH agar plates, and MBC value was defined as the lowest peptide concentration with no bacterial colony growth.

### 4.5. Time-Kinetic Inhibition Curve Assay

Overnight cultured bacteria were subcultured at a ratio of 1:100 and grown to the mid-log phase. Bacterial cells were washed three times with PBS and adjusted to 1 × 10^5^ CFU/mL, then co-incubated with antimicrobial peptides at 1 × MIC, 1/2 MIC and 1/4 MIC. A total of 100 μL of the bacteria-peptide mixture was sampled every 4 h for 24 h to determine the OD_600_ value, with three replicates for each time point.

### 4.6. Time-Kinetic Bactericidal Curve Assay

Overnight cultured bacteria were subcultured at 1:100 and grown to the mid-log phase. Bacterial cells were washed three times with PBS and adjusted to 1 × 10^5^ CFU/mL, then co-incubated with antimicrobial peptides at 1 × MBC. A total of 100 μL of the bacteria-peptide mixture was collected every 2 h for 12 h, subjected to 10-fold serial dilution and spread on agar plates for colony counting. Each time point was tested in three replicates.

### 4.7. Cytotoxicity Assay

Cells were cultured and seeded in a 96-well plate. After full confluence, antimicrobial peptides at different concentrations were added to the wells, with PBS-treated cells as the negative control. After incubation at 37 °C in a CO_2_ incubator for 2 h, the cells were washed three times with PBS, and fresh medium containing 10% CCK8 solution was added for further incubation for 3 h. The absorbance at 450 nm was measured using a spectrophotometer. Cell viability was calculated as follows: Cell viability = [(Absorbance of experimental wells − Absorbance of blank wells)/(Absorbance of control wells − Absorbance of blank wells)] × 100%.

### 4.8. Hemolysis Assay

Fresh defibrinated Sheep Red Blood Cells (SRBCs) were washed 2–3 times with PBS and centrifuged at 3000 r/min at 4 °C for 10 min. A 10% SRBC suspension was mixed with antimicrobial peptides at different concentrations, with 1% Triton X-100 as the positive hemolysis control and PBS-treated SRBCs as the negative control. The mixture was incubated at 37 °C for 6 h and then centrifuged at 2000 r/min for 10 min. The absorbance of the supernatant was measured using a multifunctional microplate reader at 540 nm.

### 4.9. Intracellular ATP Content Detection

Total and extracellular ATP contents were detected using the ATP Assay Kit was purchased by Beyotime Co., Ltd. (Shanghai, China). Bacteria were cultured overnight and subcultured to the mid-log phase, then washed three times with PBS and adjusted to an OD_600_ of 1.0. Bacterial cells were stimulated with antimicrobial peptides at different concentrations at 37 °C for 1 h, and the supernatant and precipitate were collected separately. Cell ATP lysis buffer was added to both fractions for digestion for 30 min. A total of 20 μL of the positive control and peptide-treated samples were added to a black 96-well plate, followed by the addition of 100 μL of ATP detection reagent. The luminescence value of each well was immediately measured using a multifunctional microplate reader, and the relative ATP contents were expressed as relative luminescence units (RLU).

### 4.10. Bacterial Intracellular ROS Detection

Bacteria grown to the log phase (OD_600_ ≈ 0.8) were washed three times with sterile PBS, resuspended in fresh PBS and adjusted to an OD_600_ of 0.5. The bacterial suspension was incubated with 10 μM DCFH-DA at 37 °C for 30 min. The pre-stained bacteria were washed twice with PBS, resuspended in fresh PBS, and added to a black transparent-bottom 96-well plate containing antimicrobial peptides at different concentrations, with H_2_O_2_ as the positive control (three replicates per concentration). After co-incubation for 1 h, the fluorescence intensity was measured using a multifunctional microplate reader at an excitation wavelength of 492 nm and an emission wavelength of 525 nm, and the relative ROS level was expressed as RLU.

### 4.11. Outer Membrane Permeability Assay

Overnight cultured *E. coli* ATCC25922 was subcultured at 1:100 into LB medium and grown to an OD_600_ ≈ 0.8. Bacterial cells were collected, washed three times with sterile PBS, and co-incubated with peptide solutions at different concentrations (1:1, *v*/*v*) for 2 h. A total of 630 μL of the peptide-bacteria mixture was mixed with 10 μL of 40 mM NPN dye, and 200 μL of the mixture was added to each well of a black 96-well plate (three replicates per concentration). PBS and 8 μM colistin B were used as the negative and positive controls, respectively. The fluorescence intensity was measured at an excitation wavelength of 350 nm and an emission wavelength of 420 nm using a multifunctional microplate reader.

### 4.12. Inner Membrane Permeability Assay

Overnight cultured *E. coli* ATCC25922 was subcultured at 1:100 into LB medium and grown to an OD_600_ ≈ 0.8. Bacterial cells were collected, washed three times with sterile PBS, and co-incubated with peptide solutions at different concentrations (1:1, *v*/*v*) for 2 h. A total of 630 μL of the peptide-bacteria mixture was mixed with 10 μL of 40 mM PI dye, and 200 μL of the mixture was added to each well of a black 96-well plate (three replicates per group). PBS and 8 μM colistin B were used as the negative and positive controls, respectively. The fluorescence intensity was measured at an excitation wavelength of 535 nm and an emission wavelength of 615 nm using a multifunctional microplate reader.

### 4.13. Scanning Electron Microscopy (SEM) Analysis

Bacteria were cultured overnight in LB medium at 37 °C, subcultured to the log phase, and treated with 16 μg/mL and 32 μg/mL of A7 for 1 h, then centrifuged at 4000 r/min for 10 min. The bacterial cells were washed three times with PBS, fixed with electron microscopy fixative at room temperature for 2 h and then at 4 °C overnight. After dehydration and gold sputtering coating, the bacterial morphology was observed under a scanning electron microscope.

### 4.14. Biofilm Eradication Assay

*E. coli* in the log phase was adjusted to an OD_600_ of 0.5 with LB medium, and 100 μL of the bacterial suspension was added to a 6-well plate containing cell slides. LB medium supplemented with 1% glucose was used to promote biofilm formation, and the plate was statically cultured at 37 °C for 5 d with medium change every other day. The biofilms were treated with antimicrobial peptides at different concentrations for 12 h, washed three times with PBS, and stained with SYTO 9 (green, staining all bacteria) and PI (red, staining dead/membrane-damaged bacteria) for 30 min. The slides were observed under a confocal laser scanning microscope (CLSM) for three-dimensional and serial section scanning, and images were captured and saved.

### 4.15. Tissue Bacterial Load Determination

Livers, spleens, lungs and kidneys were collected from mice in five groups on the 3rd day of the mouse protection experiment. The tissue samples were homogenized, subjected to 10-fold serial dilution, and spread on LB agar plates for colony counting. The bacterial load was expressed as colony-forming units per gram of tissue (CFU/g).

### 4.16. ELISA Detection of Inflammatory Cytokines

The levels of IL-1β and TNF-α in mouse liver tissues were detected using cytokine detection kits from Mershark Biotech Co., Ltd. (Wuhan, China). following the manufacturer’s instructions.

(1) The required microplate strips were taken out of the sealed bag; unused strips were stored at 4 °C with desiccants;
(2) Standard/sample universal diluent was added to blank wells, and 100 μL of samples or standards was added to the corresponding wells. The plate was sealed and incubated at 37 °C for 90 min;
(3) The antibody working solution was prepared 20 min in advance;
(4) The microplate was washed five times;
(5) A total of 100 μL of the antibody working solution was added to each well, sealed and incubated at 37 °C for 60 min;
(6) The HRP working solution was prepared 20 min in advance and placed at room temperature in the dark;
(7) The microplate was washed 3–5 times;
(8) 100 μL of the HRP working solution was added to each well, sealed and incubated at 37 °C for 30 min in the dark;
(9) The microplate reader was preheated, and the detection program was set;
(10) The microplate was washed 3–5 times;
(11) 100 μL of TMB chromogenic substrate was added to each well and incubated at 37 °C for 20 min in the dark;
(12) A total of 100 μL of stop solution was added to each well, and the OD_450_ value was measured immediately within 10 min;
(13) A standard curve was plotted with standard concentration as the abscissa and OD_450_ value as the ordinate, and the cytokine concentrations in samples were calculated based on the curve.

### 4.17. Histopathological Experiment

Liver and spleen samples from mice, as well as heart, liver, spleen, lung and brain samples from chicks, were fixed in 4% formaldehyde solution for 24 h after rinsing with pre-cooled PBS. The tissue blocks were sequentially dehydrated in gradient ethanol, cleared in xylene, infiltrated in paraffin wax, and embedded in paraffin blocks. Serial cross-sections (3 μm) were prepared, spread in a 42 °C water bath, and dried in a 37 °C drying oven overnight. The sections were stained with hematoxylin and eosin (H&E), mounted with neutral balsam, and observed under a light microscope. Images were collected using the Case Viewer 2.4 image processing system.

### 4.18. Ethical Statement for Animal Experiments

Specific-pathogen-free (SPF) female BALB/c mice aged 6–8 weeks were purchased from the Experimental Animal Center of Hubei Center for Disease Control and Prevention. All animal experiments were approved by the Animal Ethics Committee of Huazhong Agricultural University (Approval No.: HZAUMO-2025-0392). Mice were raised in accordance with the Animal Ethics and Care Regulations of Hubei Province.

### 4.19. Statistical Analysis

Statistical analysis was performed using GraphPad Prism 8.0 software. Experimental data were analyzed by the two-tailed unpaired *t*-test. Statistical significance was defined as: *p* < 0.05 (*), *p* < 0.01 (**), *p* < 0.001 (***), *p* < 0.0001 (****); ns indicated no statistically significant difference.

## 5. Conclusions

In this study, we engineered peptide A7 based on the type VI secretion system (T6SS) effector of APEC. A7 exhibited potent broad-spectrum activity against poultry-associated pathogens and effectively eradicated bacterial biofilms. Mechanistically, it functions through a dual mode of physical membrane disruption and ROS-mediated oxidative stress. Crucially, in vivo trials confirmed that A7 significantly improved survival rates, reduced organ bacterial burdens, and attenuated inflammation in APEC-induced sepsis models, while maintaining high biosafety. Collectively, A7 presents a promising, non-antibiotic therapeutic candidate for controlling multidrug-resistant infections in the poultry industry.

## Data Availability

The original contributions presented in this study are included in the article. Further inquiries can be directed to the corresponding authors.
